# Organic-Inorganic Artificial Ion Channel Polyvinylidene Fluoride Membranes for Controllable Selectivity Transport of Alkali Metal Cations

**DOI:** 10.3390/membranes10080174

**Published:** 2020-07-31

**Authors:** Ye Tian, Shaohua Jin, Xinxin Zhang, Lihua Wang, Yakai Lin, Yutao Jin, Lijie Li

**Affiliations:** 1 School of Material Science and Engineering, Beijing Institute of Technology, Beijing 100081, China; tian.ye@scinormem.com (Y.T.); jinshaohua@bit.edu.cn (S.J.); 2 Beijing Scinor Membrane Technology Co. Ltd., Beijing 100083, China; jinyutao@tsinghua.edu.cn; 3 Key Laboratory of Green Printing, Institute of Chemistry, Chinese Academy of Science, Beijing 100190, China; zhangxinxin8758@163.com; 4 Beijing Key Laboratory of Membrane Materials and Engineering, Department of Chemical Engineering, Tsinghua University, Beijing 100084, China

**Keywords:** artificial ion channel membrane, organic–inorganic, selectivity transport, dialysis

## Abstract

In this article, organic–inorganic hybrid materials with different functional groups were used to form organic–inorganic hybrid dense membranes for selective separation of mono/divalent ions by blending these materials and polyvinylidene fluoride (PVDF) in dimethylacetamide with HCl as the catalyst. The membranes prepared by 3-(ureido benzene) propyltriethoxysilane (H1), 3-(ureido-4-methoxyphenyl) propyltriethoxysilane (H2), 3-(ureido-3-chloro-4-methoxyphenyl) propyltriethoxysilane (H3), 3-(ureidoindazolyl) propyltrieth-oxysilane (H4), or 3-(ureidopentanol) propyltriethoxysilane (H5) were labeled as HM1–HM5, respectively. The transport properties of different chlorides were tested. The effects of different anions on sodium cation transport were also tested. The results showed that HM1–HM4 could transport monovalent Li^+^, Na^+^, and K^+^ except Ca^2+^ and Mg^2+^, and the permeability of Li^+^, Na^+^, and K^+^ through the hybrid membranes followed the order of *P*_Na+_ > *P*_K+_ > *P*_Li+_. Moreover, membranes with different H2 content were also prepared due to HM2 having the best ion transport performance. The ion transport performance increased accordingly with the mass ratio of H2 to PVDF, and the permeability of Na^+^ was twice that of Li^+^ and K^+^ when the mass ratio was 15/10. Under this condition, it was also proved that NH_4_^+^ could not transport through the hybrid membrane with various selectivity for different anions as Cl^−^ > NO_3_^−^ > HCO_3_^−^ > SO_4_^2−^.

## 1. Introduction

Artificial ion channel membranes, similar to those in living organisms in function, have attracted increasing attention due to their potential application in the fields of chemistry, biology, and materials science. Over the past decade, crown-ethers [1,2,3,4], cyclic peptides [5], and calixarene have been extensively investigated for the development of ion channel membranes through self-assembly and self-organization [6,7,8]. However, due to the complex synthesis steps of supramolecular compounds such as crown-ethers, the conditions for the assembly of these compounds into ion channels are harsh, so they are not suitable for amplification applications. Therefore, it is very important to research and develop new artificial ion channel membranes with ion transport function. 

On this basis, hybrid organic–inorganic materials offer another promising approach to designing nanostructured materials with tailored chemical and physical properties [9]. A kind of hybrid material which is represented by bridged silsesquioxanes with a wide variety of tunable properties, where the organic fragment is covalently linked to the silicate framework [10] is highly attractive. In organic–inorganic complex membranes, alkali cations (K^+^ and Na^+^) can diffuse along cation-π aromatic conduction formed in the ureidoareneheteropolysiloxane hybrids by combining the main organic aryl bridging substructure with the urea groups [11]. However, the performance of hybrid organic–inorganic membranes, that is, ion permeability and selectivity, is restricted to monovalent alkali cations, and the controllable selectivity transport of the cations was not studied [12,13]. In addition, the effect of their corresponding anions on the transport is seldom taken into consideration. Moreover, the hybrid membranes reported so far are almost constituted on a supporting layer with a multi-layer structure, complicating the research on the transport mechanism.

In this work, a simple blending method of preparation of organic–inorganic complex membranes was proposed. Polyvinylidene fluoride (PVDF) has excellent mechanical properties, high chemical resistance, good thermal stability, and excellent film-forming ability, and is widely used to separate membrane materials [14,15,16,17]. In this article, five organic–inorganic hybrid materials were used to form organic–inorganic hybrid dense membranes for selective separation of mono/divalent ions by blending these materials and polyvinylidene fluoride (PVDF) in dimethylacetamide with HCl as the catalyst. These materials were 3-(ureido benzene) propyltriethoxysilane (H1), 3-(ureido-4-methoxyphenyl) propyltriethoxysilane (H2), 3-(ureido-3-chloro-4-methoxyphenyl) propyltriethoxysilane (H3), 3-(ureidoindazolyl) propyltrieth- oxysilane (H4) and 3-(ureidopentanol) propyltriethoxysilane (H5), and the membranes prepared by them were labeled as HM1–HM5, respectively. The transport properties of different chlorides (LiCl, NaCl, KCl, NH_4_Cl, and CaCl_2_) were tested. The effects of different anions on sodium cation transport were also tested. Moreover, membranes (named M1, M2, and M3) with the different H2 contents were prepared, and the influences of the content of organic–inorganic hybrid materials on the membrane properties were also studied. 

## 2. Materials and Methods

### 2.1. Reagents and Materials

Polyvinylidene fluoride (PVDF): Solef 6010, Solvay; N, N-dimethylacetamide (DMAc) and hydrochloric acid (HCl): analytical purity (AR), Chemical Reagent Co., Ltd. of China Pharmaceutical Group, Beijing, China, the rest of the reagents were also analytical purity (AR). 

**Figure 1:**
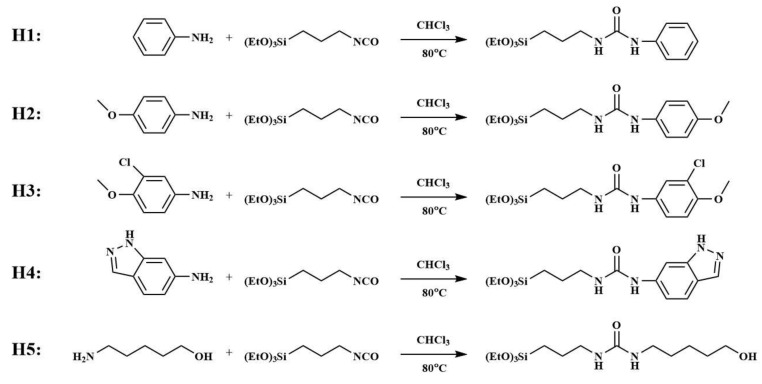
Synthesis of H1–H5.

Hybrid materials (H1–H5) were provided by the laboratory of new materials, Institute of Chemistry, Chinese Academy of Sciences, and were prepared from 3-isocyanatopropyltriethoxysilane and aniline with different functional groups, as shown in Figure 1.

### 2.2. Preparation of Hybrid Membranes with Different Hybrid Organic–Inorganic Materials

H1–H5 (5%, 0.5 g) was blended with polyvinylidene fluoride (PVDF) (15%, 1.5 g) in N, N-Dimethylacetamide (DMAC, 8 g), and 0.04 mL of 36%–37% HCl was added to the blend. The mixed solution was heated and stirred at 100 °C for 48 h to obtain a transparent membrane liquid. Then the membrane liquid was ultrasonically cleaned in an ultrasonic cleaner for 15 min, and then left for 48 h at room temperature. The corresponding hybrid membranes (HM1–HM5) were obtained by coating the solution onto a glass sheet using a spin-coating method, followed by drying under an infrared light for 15 min. For comparison, a membrane without hybrid organic–inorganic materials (M0) was also prepared.

**Figure 2:**
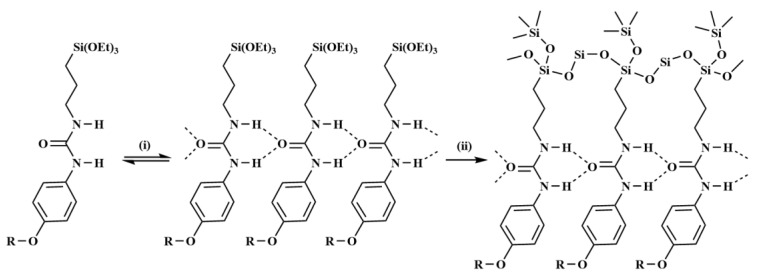
Self-organization process of organic–inorganic hybrid functional materials.

H1–H4 can self-assemble to form regular structures under neutral, acidic, or alkaline conditions, as shown in Figure 2. The (-Si(OEt)_3_) group can be hydrolyzed to form a cross-linked siloxane network (-Si-O-Si(OH)-O-Si-). At the same time, the N-H group in the molecular chain and the O on the urea group in the adjacent molecule can be bonded to each other through intermolecular hydrogen bonding to form an anion transport channel. The aromatic ring in the structure can form an aromatic channel which can transfer cations through the cation-π bond [18].

### 2.3. Preparation of Hybrid Membranes with Different Ratios of H2

By blending H2 and PVDF in three different mass ratios (1:15, 5:15, and 10:15) in dimethylacetamide with HCl as the catalyst and heating at 100 °C for 48 h, a transparent and homogeneous solution was formed. The corresponding hybrid membranes (M1, M2, and M3) were obtained by coating the solution onto a glass sheet using a spin-coating method, followed by drying under an infrared light for 15 min. The proportion of the materials in the membrane solution is shown in Table 1.

### 2.4. Characterization of Hybrid Membranes

#### 2.4.1. Observation of the Morphology of the Hybrid Membranes

The surface and cross-section of the membranes were observed by a scanning electron microscope (SEM, Hitachi S-4800, Tokyo, Japan) with the accelerating voltage set to 1.0 kV. The membranes were fractured in liquid nitrogen and coated with platinum for the SEM cross-section structure observation. 

In order to further observe the morphology of the organic–inorganic hybrid ion channel membrane, the thin film samples were prepared by ion thinning technology, and then were directly observed by a transmission electron microscope (TEM, JEOL 2010F, Tokyo, Japan) with the accelerating voltage set to 200 kV. 

#### 2.4.2. Dialysis Transport Procedure

A membrane transport experiment was performed with a bi-compartment device [19]. The device consists of two cells (the volume of each cell is 1 L) separated by a solid membrane (S = 0.5024 cm^2^) with the active dense film facing the feed phase. The feed phase was an aqueous solution containing either 0.3 M CaCl_2_, 0.3 M MgCl_2_, or 0.3 M LiCl + NaCl + KCl for the cation transport experiments, while the receiving phase contained only deionized water with the same volume. The electrical conductivity and the concentrations of Li^+^, Na^+^, K^+^, and Ca^2+^ were monitored at different time intervals by using a conductivity analyzer and a flame photometer. This experiment was carried out under the static state.

The transport performance of the membranes was also further evaluated by measuring the ionic current across the membranes in the environment with different chloride salts and sodium salts. Ionic current measurement was carried out with a Keithley 6487 picoammeter and custom designed electrolyte cells with the membrane mounted in between. When the measurement was in operation, the same kind of salt solution with the same volume and concentration (0.1 mol/L) was in the cells on either side of the membrane and the micro voltage and current were recorded by the picoammeter.

## 3. Results and Discussions

### 3.1. Microstructure and Morphology of Hybrid Membranes 

**Figure 3:**
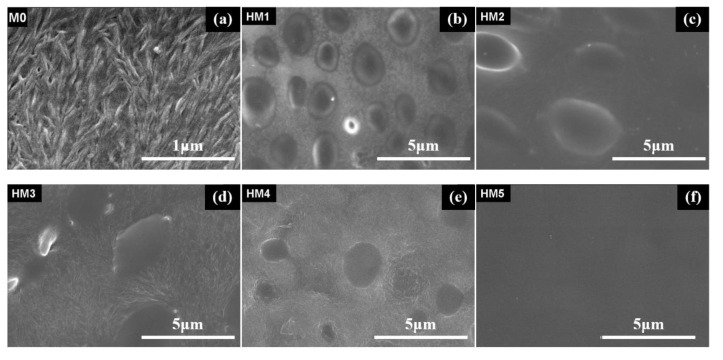
SEM images of the surfaces of the blank membrane and organic–inorganic hybrid ion channel membranes: (**a**) M0; (**b**) HM1; (**c**) HM2; (**d**) HM3; (**e**) HM4; (**f**) HM5.

**Figure 4:**
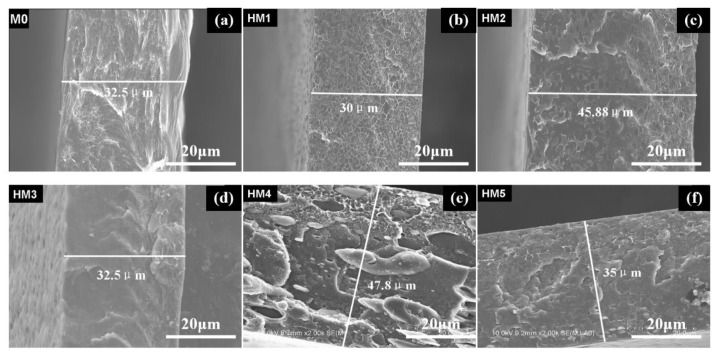
SEM images of the cross-sections of the blank membrane and organic–inorganic hybrid ion channel membranes: (**a**) M0; (**b**) HM1; (**c**) HM2; (**d**) HM3; (**e**) HM4; (**f**) HM5.

Figure 3 and Figure 4 show the microstructures and morphologies of M0 and HM1–HM5. It can be seen that the surfaces of these membranes are dense without micropores, and the thicknesses of the membranes are about 30–48 μm. Moreover, there is a dendrite crystal structure on the surface of M0, but the membrane surface is dense without micropores, and the film thickness is 32.5 μm. Two kinds of structures can be observed on the surfaces of HM1–HM4: one is the dendritic crystal structure, and the other is the smooth discontinuous elliptical structure. The elliptical structure is self-organically formed in the sol-gel process of organic–inorganic hybrid ion channel in the functional material H1–H4, which is consistent with the cross-section structure of (b), (c), (d), and (e) in Figure 4. The surface structure of HM5 is smooth and dense, which is quite different from that of the HM1–HM4 membranes. This is due to the existence of a linear pentanol group in the structure of functional material H5, which has good compatibility with PVDF, and there is no obvious two-phase structure when the two materials are mixed into the membrane.

**Figure 5:**
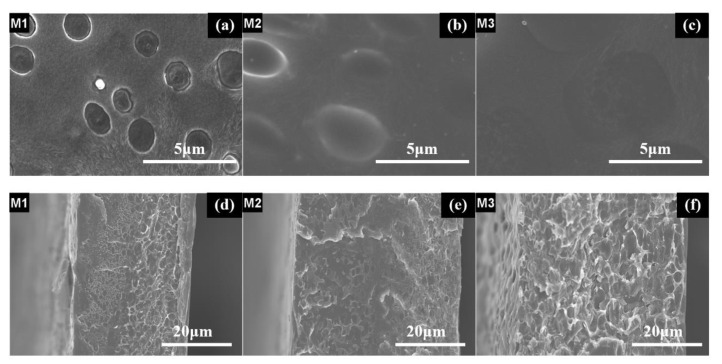
SEM images of the surfaces of M1 (**a**), M2 (**b**), M3 (**c**), and the cross-sections of M1 (**d**), M2 (**e**), M3 (**f**).

As shown in Figure 5, scanning electron microscopy (SEM) reveals that the membranes are dense without micro pinholes. Figure 3a and Figure 4a depict the surface and the cross-section of PVDF blank membrane M0 and show a well-resolved bulk crystalline area at nanometric scale. With the addition of H2, some oval-shaped domains from H2 were clearly observed in the matrix, as shown in Figure 5a for M1. With the further increase of H2 content in the membrane, the domains from H2 spread and made contact with each other to form a smooth oval-shaped structured membrane, which can also be observed in the cross-section images of M1, M2, and M3 (Figure 5d–f), as compared to that of the M0. 

**Figure 6:**
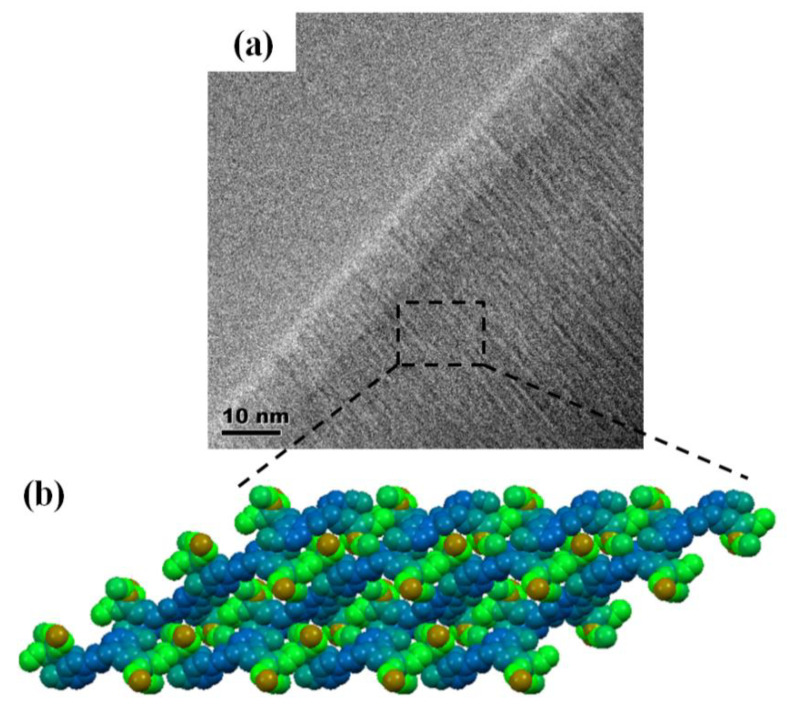
Progressive nanoscale self-organization of the hybrid membrane: (**a**) TEM image of the hybrid membrane M3; (**b**) the crystal packing of compound H2.

Figure 6a depicts a typical TEM image of an M3 membrane and shows well-resolved and highly ordered uniform rows. The repeating motif is formed by discrete alternative light (inorganic siloxane matrix) and dark (organic self-assembled molecules) rows. The periodicity of parallel sheets of the self-organized organic and siloxane inorganic networks are observed in the crystal packing (Figure 6b), which is consistent with the structure observed in the TEM.

### 3.2. Dialysis of the Transport Properties of the Membranes

**Figure 7:**
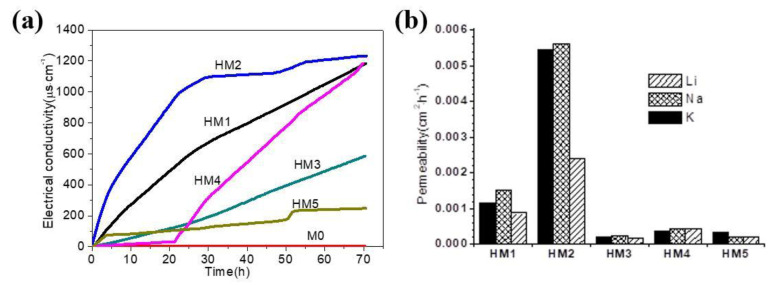
(**a**) Electrical conductivity of Li+, Na+, and K+ in M0 and HM1–HM5; (**b**) permeability values of Li+, Na+, and K+ cations in HM1–HM5.

Figure 7 shows the time-dependent curve of the conductivity of Li^+^, Na^+^, and K^+^. It can be seen from Figure 7a that the conductivity of M0 remains basically unchanged, indicating that PVDF has no transport performance for Li^+^, Na^+^, and K^+^. The electrical conductivity of HM1, HM2, and HM4 increased rapidly, and the conductivity increased by about 1000 μs/cm within 70 h. The conductivity of HM3 and HM5 increased slowly, only increasing by about 200–500 μs/cm at 70 h. The experimental results show that the organic–inorganic hybrid ion channel functional materials HM1–HM5 have certain transport properties for Li^+^, Na^+^, and K^+^, and among H1–H5, H2 has the best ion transport performance. 

Figure 7b shows the permeability values of Li^+^, Na^+^, and K^+^ of HM1–HM5, which were calculated through the reported method [19,20]. It can be seen that the permeability of Li^+^, Na^+^, and K^+^ in HM1, HM2, and HM3 is *P*_Na+_ > *P*_K+_ > *P*_Li+_. The permeability of Li^+^, Na^+^, and K^+^ in HM4 is *P*_Na+_ ≈ *P*_K+_ > *P*_Li+_, and that of HM5 is *P*_K+_ > *P*_Na+_ ≈ *P*_Li+_. In addition, it also can be seen from Figure 7b that the permeability of Li^+^, Na^+^, and K^+^ of HM2 is much higher than that of the other four membranes (H2 > H1 > H4 > H3 > H5).

The aromatic ring in the structure of H1–H4 can form an aromatic channel which can transfer cations through the cation-π bond. Therefore, the ionic transport properties depend on the cation-π interaction between cations and benzene or heterocycles. The electron donating group can strengthen the cation-π force, while the electron withdrawing group will weaken the cation-π force. In H2, the methoxy (-OCH_3_) on the para substituent of the benzene ring is an electron donating group, which can increase the cation-π interaction between Li^+^, Na^+^, and K^+^ and the benzene ring, thus promoting the ion transport of the HM2 membrane. In H3, the chlorine group (-CL) on the substituent of the benzene ring is an electron withdrawing group, and its electronegativity is greater than that of methoxy (-OCH_3_) on the para substituent. Therefore, the substituent on the benzene ring acts as an electron withdrawing group, which inhibits the ion transport of HM3. In H4, although the two adjacent N heteroatoms on the indazole group make the induced electron absorption capacity of the group a bit larger and inhibit the ion transport of HM4, the electron absorption ability of H4 is still weaker than that of H3, resulting in better ion transport performance in HM4 than in HM3. In H1, there is no substituent in the benzene ring, so the ion transport capacity of HM1 is less than that of HM2, but higher than that of HM3 and HM4. It also can be seen that HM5 has the worst transport performance for Li^+^, Na^+^, and K^+^, which indicates that the pentanol group in the straight chain has no effect on ion transport.

**Figure 8:**
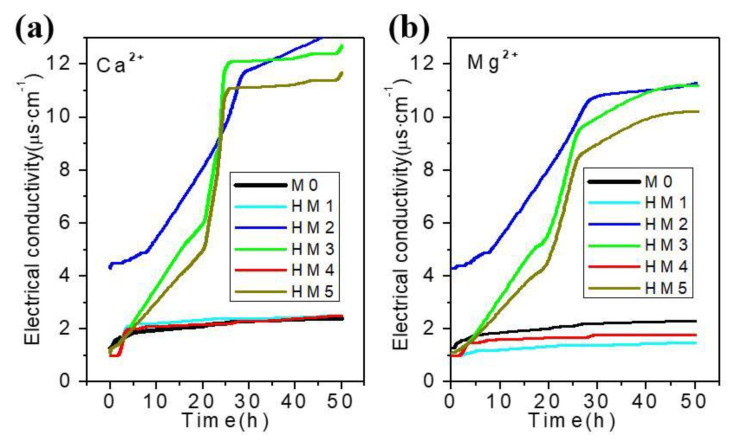
Electrical conductivity of Ca^2+^ (**a**) and Mg^2+^ (**b**) in blank membrane (M0) and organic–inorganic hybrid ion channel membranes (HM1–HM5) as a function of time.

Figure 8 shows the curve of conductivity of Ca^2+^ and Mg^2+^ with time. It can be seen from Figure 8 that the conductivity of Ca^2+^ and Mg^2+^ of M0, HM1, and HM4 remains unchanged, while the conductivity of HM2, HM3, and HM5 increases, but the conductivity only increases about 10 μs/cm in 50 h, which can be ignored. The results show that PVDF and HM1–HM5 have no transport properties for Ca^2+^ and Mg^2+^.

**Figure 9:**
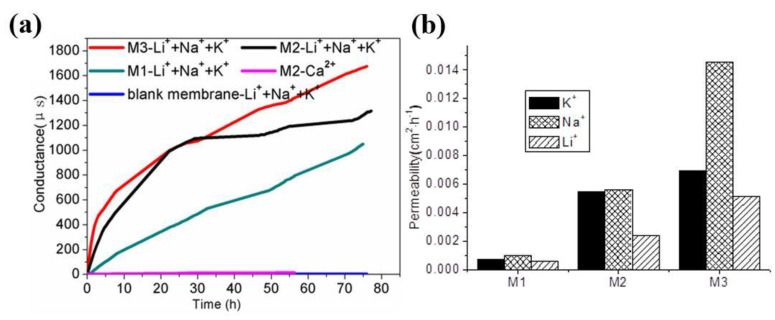
(**a**) Conductance profiles in the strip phase as a function of time; (**b**) permeability values of Li^+^, Na^+^, and K^+^ cations in the M1, M2, and M3 hybrid membranes.

Figure 9a shows the conductance change of M0, M1, M2, and M3 with time for Li^+^, Na^+^, K^+^, and Ca^2+^. With the addition of H2, the conductance increases for all the cations with increasing the content of H2 in the membranes and presents a similar increasing trend. Thus, transport behavior was significantly dependent on the ratio of H2 and PVDF in the membranes. Conductance for Ca^2+^ was near zero with M2, despite the fact that the high conductance was measured for Li^+^, Na^+^, and K^+^ with it. Those results proved that H2 could transport Li^+^, Na^+^, and K^+^, but was not selective for Ca^2+^. The permeabilities of Li^+^, Na^+^, and K^+^ followed the order *P*_Na+_ > *P*_K+_ > *P*_Li+_ and were amplified when the content of H2 increased in the membrane (Figure 9b). However, there was little difference among the permeabilities of Li^+^, Na^+^, and K^+^ in both M1 and M2. When the ratio of H2 and PVDF increased to 10:15 in M3, the difference was remarkably enlarged, as shown in Figure 9b, and the permeabilities of Na^+^ was twice more than that of Li^+^ or K^+^. The results revealed that H2 is well selected for Na^+^ transport. Usually, Li^+^, Na^+^, and K^+^ transport through the membranes in the forms of hydrated cations, and hydrated ionic radii follow the order Li(H_2_O)_n_^+^ > Na(H_2_O)_n_^+^ > K(H_2_O)_n_^+^, that is, Li(H_2_O)_n_^+^ is disadvantaged in membrane transport. 

**Figure 10:**
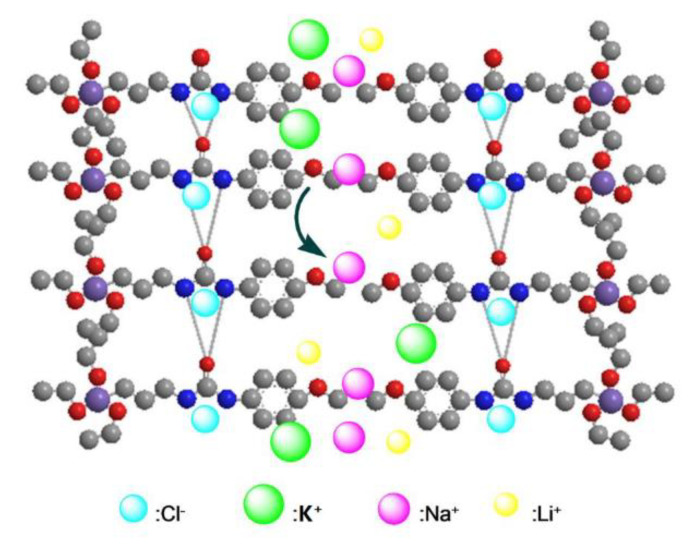
Aromatic cation-π and urea anion conduction pathways.

A substantial contribution to Na^+^ transport selectivity through M3 is due to the interaction of the hydrated cations with the hydrophilic methoxyphenyl pathway (Figure 10). The benzene ring could effectively compete with water in the solvation sphere of K^+^ leading to the formation of mixed K^+^/Ar_x_/(H_2_O)_y_ clusters, which means that partially dehydrated K^+^ may stick within the methoxyphenyl pathway of the membrane [9]. It has been proven that K^+^ interacts with the phenyl in an aqueous solution whereas Na^+^ cation does not [21,22]. Therefore, the stronger interaction of Na^+^ with water prevents cation dehydration, and thus Na^+^ cation is selectively transported as hydration species Na(H_2_O)_n_^+^ through the hydrophilic pathways of M3.

**Figure 11:**
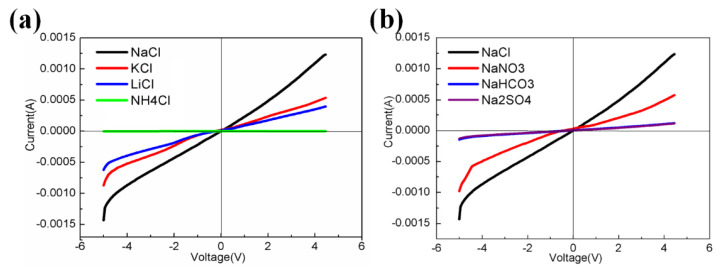
Current–voltage properties of membrane M3: (**a**) different transported cations with the same anion Cl^−^, (**b**) different transported anions with the same cation Na^+^.

The transport performance of M3 was evaluated by measuring the ionic current across the membrane in an environment with different chloride salts and sodium salts. Ionic current measurement was carried out with a Keithley 6487 picoammeter in a custom designed electrolyte cell with the membrane mounted in between. Figure 11 shows current–voltage (I–V) curves of M3. Figure 11a compares the transmembrane currents of the monovalent salts of NaCl, KCl, LiCl, and NH_4_Cl. The currents of NaCl, KCl, and LiCl kept an increasing trend with increasing the applied voltages, and the current of NaCl was the highest among them, consistent with the conductance measured above. From Figure 11a, it is clear that the current of NH_4_Cl kept near zero with the applied voltages, indicating that M3 cannot transport NH_4_^+^. For further investigation of anion effect on the transport, a series of sodium salts (NaCl, NaNO_3_, NaHCO_3_, and Na_2_SO_4_) were selected to measure the ionic currents. As shown in Figure 11b, the chloride passed through the membrane faster than the nitrate, bicarbonate or sulfate. This result can be explained due to the migration rates of the anions. When the sodium salts have an equal number of moles, the migration rates of anions follow the order Cl^−^ > NO_3_^−^ > HCO_3_^−^ > SO_4_^2−^. The membrane has double channels, and can transport ion pairs. The transport rates of cations and anions would influence each other, and the selectivity for Na^+^ strongly relates to the type of the anions.

## 4. Conclusions

In this article, H1–H5 organic–inorganic hybrid materials were used to form organic–inorganic hybrid dense membranes. The aromatic ring in the structure of H1–H4 can form an aromatic channel which can transfer cations through the cation-π bond. In H2, the methoxy (-OCH_3_) on the para substituent of the benzene ring is an electron donating group, which can increase the cation-π interaction between Li^+^, Na^+^, and K^+^ and the benzene ring, thus promoting the ion transport performance of the HM2 membrane. With the increase of H2, the ion transport performance of the hybrid membrane increased accordingly. When the mass ratio of H2 to PVDF was 15:10 (M3), the permeability of Na^+^ was twice than that of Li^+^ and K^+^, which showed a good selectivity for Na^+^. The results of I–V curves showed that NH_4_^+^ could not transport through M3 and M3 had various selectivity for different anions, such as Cl^−^ > NO_3_^−^ > HCO_3_^−^ > SO_4_^2−^, suggesting that M3 was well selected for Cl^−^. The hybrid membranes could find application in the softening of hard water, the desalination of sea water, ion selection electrodes, biosensors, and other fields. 

## Figures and Tables

**Table 1:** The proportion of the materials in the membrane solution.

Materials	M1	M2	M3
PVDF(15%)/g	1.5	1.5	1.5
DMAC/g	8.4	8	7.5
H2/g	0.1(1%)	0.5(5%)	1(10%)
HCl/mL	0.02	0.04	0.08
